# Time series analysis and forecasting with ECOTOOL

**DOI:** 10.1371/journal.pone.0221238

**Published:** 2019-10-31

**Authors:** Diego J. Pedregal

**Affiliations:** ETSI Industriales, Universidad de Castilla-La Mancha, Ciudad Real, Spain; Tongii University, CHINA

## Abstract

This paper presents ECOTOOL, a new free MATLAB toolbox that embodies several routines for identification, validation and forecasting of dynamic models. The toolbox includes a wide range of exploratory, descriptive and diagnostic statistical tools with visual support, designed in easy-to-use Graphical User Interfaces. It also incorporates complex automatic procedures for identification, exact maximum likelihood estimation and outlier detection for many types of models available in the literature (like multi-seasonal ARIMA models, transfer functions, Exponential Smoothing, Unobserved Components, VARX). ECOTOOL is the outcome of a long period of programming effort with the aim of producing a user friendly toolkit such that, just a few lines of code written in MATLAB are able to perform a comprehensive analysis of time series. The toolbox is supplied with an in-depth documentation system and online help and is available on the internet. The paper describes the main functionalities of the toolbox, and its power is shown working on several real examples.

## Introduction

The rapid development of Information and Communication Technologies has open the door to the use of massive amounts of data in virtually any area of science and industry. When trying to forecast such amount of information, there is an imperative need for methods and tools capable of providing automatic, general, efficient and reliable solutions in many different contexts.

One of such tools for time series analysis and forecasting is ECOTOOL, a new MATLAB toolbox introduced in this paper. It includes routines for well-know methods, like regression, ARIMA(X), Transfer Functions, VAR(X), ExponenTial Smoothing (ETS), but it also includes less common methods, mainly Unobserved Components models (UC). It offers abundant descriptive and diagnostic statistics in friendly Graphical User Interfaces (GUI), automatic procedures of identification and outliers detection, auxiliar functions for building calendar typical intervention variables, additional tools to make routine operations easy and a long list of demos.

There are some commercial and open-source pieces of software similar and complementary to ECOTOOL: Econometrics and GARCH official MATLAB toolboxes [1], CAPTAIN [2], SCA [3], TRAMO/SEATS [4], forecast package in R [5], gretl [6], STAMP [7], Eviews [8], SAS [9], Stata [10], etc.

ECOTOOL gathers in a single toolbox many flexible features scattered among other packages that served as inspiration. Many users might find similarities with other pieces of software, mainly CAPTAIN, SCA, TRAMO/SEATS and the forecast package. Most of such similarities have to do with visualisation interfaces, treatment of missing data, automatic identification methods, outliers detection procedures, diagnostic testing, etc.

But, despite all the similarities with previous work, there are some unique features of ECOTOOL that very rarely are found elsewhere, to the author knowledge. Six most important are: i) automatic identification routines for multi seasonal ARIMA models; ii) estimation of UC and ETS models with inputs modeled as linear transfer functions (also linear regressions, as they are particular cases of transfer functions); iii) estimation of most models by Exact Maximum Likelihood, this is usual for many types of models, but rather unusual for ETS, see e.g. [11]; iv) automatic outliers detection in UC and ETS models; v) particular handy ways to specify transfer function and ARIMA models, since they are directly passed to ECOTOOL as string variables very similar to the analytical expression written on a paper (see the Toolbox overview section); vi) forecasting transfer function models with stochastic inputs taking into account the uncertainty around univariate forecasts of input variables.

Further advantages of ECOTOOL, shared with other packages, is that it is integrated with the rest of toolboxes available in MATLAB as a unified environment and it is freely available at https://github.com/djpt999/ECOTOOL.

The methods implemented in ECOTOOL have potential applications in many areas of research, from economics to engineering, including many other areas related to life and social sciences or environmental sciences, see e.g., [2, 12–19]

The rest of the paper is organised as follows. Next section shows the main methods available in ECOTOOL. Then, a quick guide to the most important features of the toolbox is presented. Afterwards, several real examples are discussed in depth to show the capabilities of ECOTOOL. Finally, the paper concludes with some relevant remarks.

## Models and methods implemented in ECOTOOL

ECOTOOL offers the possibility of identifying, estimating, testing and forecasting time series by methods with different degrees of complexity, from pure univariate to full multivariate systems. The next sub-sections show the full list of methods implemented.

### Naïve methods

The simplest methods, commonly used as benchmarks, are a set of naïve methods, i.e., mere rules of thumb that needs just simple computations or no computations at all. They are strictly correct under some severe restricting assumptions, but usually most of them have no meaning at all for many time series.

Let’s call *y*_*t*_, *t* = 1, 2, …, *T* a time series; *s* the seasonal period; *l* the forecasting horizon and ⌊*x*⌋ the integer part of *x*. Then, the *l* steps ahead forecasts conditional on all information available up to time *T* (i.e., ${\hat{y}}_{T+l}$) is estimated by each of the naïve methods as shown in Table 1. It is assumed in general that if more sophisticated methods are better in forecasting terms, they should outperform all these naïve options. *Mean* forecasts are just the mean of the in-sample data; *RW* forecasts are just the last observation propagated into the future; *seasonal RW* is the repetition of the last seasonal cycle available; *mean seasonal RW* is the mean of the last seasonal cycle of data; *drift* is a linear forecast with a slope calculated by joining the last and the first observations; *mean drift* is similar but the slope is based on the *mean seasonal RW* forecast.

**Table 1:** Naïve methods implemented in function modelNAIVE.

Method	Forecast calculation (${\hat{y}}_{T+l}$)
*Mean*	$\sum_{t=1}^{T}y_{t}/T$
*Random Walk* (RW)	*y*_*T*_
*Seasonal RW*	*y*_*T*+*l*−(⌊(*l*−1)/*s*⌋+1)*s*_
*Mean Seasonal RW*	$\sum_{k=l-s+1}^{l}{\hat{z}}_{T+k}$, where ${\hat{z}}_{T+k}$ are the *seasonal RW* forecasts
*Drift*	*y*_*t*_ + (*y*_*T*_ − *y*_1_) × *l*/(*T* − 1)
*Mean Drift*	*y*_*t*_ + (*x*_*T*_ − *y*_1_) × *l*/(*T* − 1), where *x*_*T*_ is the mean of the data in the last season available

All these naïve methods are implemented in the function modelNAIVE.

### ARIMA

The ARIMA models implemented in ECOTOOL in the function modelTF are rather general [12], since it allows for multiple seasonal polynomials. This is what some authors have called multi-seasonal ARIMA models. The general formulation is in Eq (1), where *z*_*t*_ is a stationary time series; *B* is the back-shift operator such that *B*^*l*^ *z*_*t*_ = *z*_*t*−*l*_; $\theta_{Q_{i}}\left(B^{s_{i}}\right)$ and $\phi_{P_{i}}\left(B^{s_{i}}\right)$ are moving average and autoregressive polynomials of order *Q*_*i*_ and *P*_*i*_, respectively, in which the exponent of the backshift operator in each summand is a multiple of the seasonal frequency, *s*_*i*_ (*i* = 1, 2, …, *k*); and *a*_*t*_ is a Gaussian serially independent white noise with zero mean and constant variance.

$$\begin{array}{c} z_{t}=\frac{\theta_{Q_{0}}\left(B\right)}{\phi_{P_{0}}\left(B\right)}\frac{\theta_{Q_{1}}\left(B^{s_{1}}\right)}{\phi_{P_{1}}\left(B^{s_{1}}\right)}\frac{\theta_{Q_{2}}\left(B^{s_{2}}\right)}{\phi_{P_{2}}\left(B^{s_{2}}\right)}\cdots \frac{\theta_{Q_{k}}\left(B^{s_{k}}\right)}{\phi_{P_{k}}\left(B^{s_{k}}\right)}a_{t} \end{array}$$ (1)

Time series *z*_*t*_ in Eq (1) is assumed stationary, but usually is the result of applying the differencing operators to a non stationary time series *y*_*t*_, as in Eq (2).

$$\begin{array}{c} z_{t}={\left(1-B\right)}^{d_{0}}{\left(1-B^{s_{1}}\right)}^{d_{1}}\ldots {\left(1-B^{s_{k}}\right)}^{d_{k}}y_{t} \end{array}$$ (2)

ECOTOOL offers an automatic identification procedure inspired in [5] applied to multi seasonal ARIMA models. It is effectively a much more complex process than [14] in the sense that identification is fully automatic and that it allows for moving average terms. As far as the author is concerned, this is the first time such identification procedure is implemented successfully in a time series package for multi seasonal models (see the ‘Case studies’ section below).

### Unobserved Components (UC)

ECOTOOL allows for UC models known as a Basic Structural Model of Harvey in the function modelUC [13]. The formulation of this model is shown in Eq (3), where a time series *y*_*t*_ is decomposed into a long term trend (*T*_*t*_), a seasonal component (*S*_*t*_) and an irregular component (*I*_*t*_).

$$\begin{array}{c} y_{t}=T_{t}+S_{t}+I_{t} \end{array}$$ (3)

Particular formulations are possible by selecting different alternatives of each component. For trend models, the possibilities are condensed in Eq (4), where $T_{t}^{*}$ is referred to as the trend ‘slope’, *α* is a parameter between zero and one, and *η*_*t*_ and $\eta_{t}^{*}$ are independent white noise sequences with variances $\sigma_{\eta }^{2}$ and $\sigma_{\eta^{*}}^{2}$, respectively.

$$\begin{array}{c} \left[\begin{array}{c} T_{t+1}\\ T_{t+1}^{*} \end{array}]=[\begin{array}{cc} \alpha & 1\\ 0 & 1 \end{array}][\begin{array}{c} T_{t}\\ T_{t}^{*} \end{array}]+[\begin{array}{c} \eta_{t}\\ \eta_{t}^{*} \end{array}\right] \end{array}$$ (4)

This model is called Generalised Random Walk Trend and subsumes the following specific cases implemented in ECOTOOL: i) Random Walk (RW), by eliminating the second equation and *α* = 1; ii) Integrated Random Walk (IRW) with *α* = 1 and $\sigma_{\eta }^{2}=0$; iii) Smoothed Random Walk (SRW) with $\sigma_{\eta }^{2}=0$; and iv) Local Linear Trend (LLT) with *α* = 1, see e.g., [13], [2] and [20].

Seasonal components are included as the so called dummy seasonality [13], that depends on a single parameter, namely the variance of white noise *ω*_*t*_, see Eq (5).

$$\begin{array}{c} \begin{array}{c} \left[\begin{array}{c} S_{1,t+1}\\ S_{2,t+1}\\ S_{3,t+1}\\ \vdots \\ S_{s-1,t+1} \end{array}]=\Psi [\begin{array}{c} S_{1,t}\\ S_{2,t}\\ S_{3,t}\\ \vdots \\ S_{s-1,t} \end{array}]+[\begin{array}{c} \omega_{t}\\ 0\\ 0\\ \vdots \\ 0 \end{array}\right]\\ \text{with}\Psi =[\begin{array}{ccccc} -1 & -1 & -1 & \cdots & -1\\ 1 & 0 & 0 & \cdots & 0\\ 0 & 1 & 0 & \cdots & 0\\ \vdots & \vdots & \vdots & \ddots & \vdots \\ 0 & 0 & 0 & \cdots & 0 \end{array}] \end{array} \end{array}$$ (5)

The full UC model is built by assembling all the State Space models for the components. As a matter of fact, Eq (3) is the observation equation of a State Space system and the block concatenation of all models for the trend in Eq (4) and seasonal in Eq (5) form the transition equation. The Kalman Filter and other recursive algorithm provides the optimal solution to state estimation, see details in [2, 13, 20].

### ExponenTial Smoothing (ETS)

The ETS models implemented in function modelETS are taken from [11], see Table 2 for the full list of possibilities. Multiplicative forms are possible by using the log transformation.

**Table 2:** ETS type of components in modelETS.

Code	Model
NN	*y*_*t*_ = *l*_*t*−1_ + *e*_*t*_
	*l*_*t*_ = *l*_*t*−1_ + *αe*_*t*_
AN	*y*_*t*_ = *l*_*t*−1_ + *b*_*t*−1_ + *e*_*t*_
	*l*_*t*_ = *l*_*t*−1_ + *b*_*t*−1_ + *αe*_*t*_
	*b*_*t*_ = *b*_*t*−1_ + *βe*_*t*_
DN	*y*_*t*_ = *l*_*t*−1_ + *b*_*t*−1_+ *e*_*t*_
	*l*_*t*_ = *l*_*t*−1_ + *b*_*t*−1_ + *αe*_*t*_
	*b*_*t*_ = *ϕb*_*t*−1_ + *βe*_*t*_
NA	*y*_*t*_ = *S*_*t*−*s*_ + *e*_*t*_
	*S*_*t*_ = *S*_*t*−*s*_ + *γe*_*t*_
AA	*y*_*t*_ = *l*_*t*−1_ + *b*_*t*−1_ + *S*_*t*−*s*_ + *e*_*t*_
	*l*_*t*_ = *l*_*t*−1_ + *b*_*t*−1_ + *αe*_*t*_
	*b*_*t*_ = *b*_*t*−1_ + *βe*_*t*_
	*S*_*t*_ = *S*_*t*−*s*_ + *γe*_*t*_
DA	*y*_*t*_ = *l*_*t*−1_ + *b*_*t*−1_ + *S*_*t*−*s*_ + *e*_*t*_
	*l*_*t*_ = *l*_*t*−1_ + *b*_*t*−1_ + *αe*_*t*_
	*b*_*t*_ = *ϕb*_*t*−1_ + *βe*_*t*_
	*S*_*t*_ = *S*_*t*−*s*_ + *γe*_*t*_

In Table 2, *l*_*t*_, *b*_*t*_, *S*_*t*_ and *e*_*t*_ stand for the level, slope, seasonal and irregular components respectively; and *α*, *β*, *γ* and *ϕ* are unknown parameters that should be estimated. The code is composed of two letters and defines the model components, the first letter specifies the trend and the second letter is reserved for the seasonal component. ‘N’ indicates that the component is not present; ‘A’ implies that the component is additive; ‘D’ implies a damped component, applicable to trends only (with 0 < *ϕ* < 1). When specifying an ETS model with a seasonal component, the two letters ought to be followed by a number indicating the fundamental period in samples per cycle.

### Transfer function (TF)

Multiple Input Single Output TF models are implemented in ECOTOOL by means of the modelTF function. The model may be written as in Eq (6).

$$\begin{array}{c} y_{t}=\sum_{i=1}^{h}\frac{\omega_{n_{i}}\left(B\right)}{\delta_{m_{i}}\left(B\right)}u_{i,t}+N\left(B\right)a_{t} \end{array}$$ (6)

In this equation, $\omega_{n_{i}}\left(B\right)=\left(\omega_{0}+\omega_{1}B+\omega_{2}B^{2}+\cdots +\omega_{n_{i}}B^{n_{i}}\right)$ and $\delta_{m_{i}}\left(B\right)=\left(1+\delta_{1}B+\delta_{2}B^{2}+\cdots +\delta_{m_{i}}B^{m_{i}}\right)$ are polynomials in the backshift operator of order *n*_*i*_ and *m*_*i*_, respectively; and *N*(*B*)*a*_*t*_ is a general representation for any of the univariate alternatives in ECOTOOL, namely ARIMA, UC, and ETS. Mind that linear regression is a particular case of model (6) if all numerator and denominator polynomials are of order zero.

When the noise model in Eq (6) is specified as an AR, ARMA or ARIMA model, it is possible to transform the TF model into an ARX, ARMAX or ARIMAX, by setting appropriate constraints on the polynomials. This operation is straightforward in ECOTOOL due to the way the models are specified (see section Case studies).

While linear regression or TF models with ARIMA noise have been used abundantly for a long time since the first edition of [12] in 1970, ETS or UC combined with TF models is a unique feature of ECOTOOL toolbox, as far as the author is concerned. Taking advantage of this feature, function modelTF also allows for the automatic detection of four types of outliers in ARIMA, UC and ETS models (see Case studies section). Once more, automatic identification of outliers in UC or ETS models is a unique feature of ECOTOOL.

### VARX

VARX models in Eq (7) are the multivariate option implemented in ECOTOOL by means of the function modelVARX, where boldface letters indicate either matrices or vectors.

$$\left(I+\Phi_{1}B+\cdots +\Phi_{p}B^{p}\right)z_{t}=\left(\Omega_{0}+\Omega_{1}B+\cdots +\Omega_{q}B^{q}\right)u_{t}+a_{t}$$ (7)

In this equation, **z**_*t*_ stands for a vector of *p* stationary outputs (generally obtained by appropriate differencing of corresponding vector of non-stationary time series **y**_*t*_); **u**_*t*_ represent a set of *m* inputs; **Φ**_*i*_ are a set of squared matrices of dimension *p* × *p*; **Ω**_*j*_ are a set of matrices of dimension *p* × *m*; and **a**_*t*_ are a vector of *p* white noises with zero mean and non-diagonal covariance matrix **Γ**.

Optimal estimation of unconstrained VARX models is easy, since equation by equation estimation by least squares renders consistent and efficient estimates. ECOTOOL allows for imposing constraints on the coefficients for which iterative generalised least squares are used to reach efficient estimation with a number of iterations controlled by the user [16].

## Toolbox overview

ECOTOOL is user oriented in the sense that the coding effort demanded from the user is reduced to a minimum at the cost of the programmer elaborating long and comprehensive functions. The result is that it is possible to carry out an exhaustive analysis of time series with the recourse to a few functions. The main ones are listed on Table 3 in separated sections showing the GUI and demo tools, model functions, functions for the generation of calendar effects dummies and general purpose functions. The real number of functions in ECOTOOL is actually much greater because all the available options in the menus of the toolTEST GUI are actually implemented as separate functions that may be run independently, see the documentation. Some explanations about this functions are included in the next paragraphs and the way to use them is illustrated in the worked examples below.

**Table 3:** Main ECOTOOL functions.

GUI and demos	
toolTEST	Exploratory, descriptive and diagnostic checking tool
toolFORECAST	Forecasting tool
ECOTOOLdemos	ECOTOOL demos
Modeling functions	
modelAUTO	Automatic identification of ARIMA models
modelETS	Exponential Smothing models
modelNAIVE	Forecasting with several naive models
modelTF	MISO Transfer Function analysis
modelUC	Unobserved Components models
modelVARX	VAR model with eXogenous variables analysis
Calendar effects	
days	Dummy variable for number of days in months or quarters
easter	General dummy Easter variables on monthly or quarterly data
leapy	Dummy variable for leap year intervention
trading	Trading day variables on monthly or quarterly frequency
General purpose functions	
acft	Theoretical autocorrelation functions of ARMA processes
varstep	Var impulse and step function analysis
vboxcoxinv	Inverse of Box-Cox transformation
vConv	Multiplication of vector polynomials
vdif	Differenciation of a vector of variables
vfilter	Filters a vector of inputs with a vector digital filter
vRoots	Roots of a vector polynomial

The main features of ECOTOOL are:

- As shown in the previous section, a number of time series methods are implemented, ranging from simple naïve univariate models to multivariate methods.
- Extensive and detailed documentation is available. All functions are provided with a thorough help accessible in the usual way. Eleven detailed demos with extensive explanations that show all the properties of the toolbox are also provided (accessed by ECOTOOLdemos).
- The design of the toolbox is such that it is possible to perform a full time series analysis with just a few MATLAB instructions. In this way, the memory effort demanded from the user is reduced to a minimum. In addition, function names are selected following mnemonic rules such that they are easy to remember and easy to look for.
- The toolbox is composed of four types of functions, see Table 3: i) GUI tools to perform several tasks that are named as tool* (where ‘*’ stands for a name, at the moment two are available, toolTEST and toolFORECAST, see below); ii) Modeling methods that are named as model* (like modelNAIVE, modelTF, etc.); iii) functions that facilitates building dummy variables to deal with calendar effects; and iv) general purpose functions to perform a number of important tasks when dealing with time series analysis, like transforming the data (standardising, differencing, Box-Cox variance transforming, etc.) or doing other tasks (filtering and differencing vector time series, calculating convolutions of multivariate polynomials, calculating roots of multivariate polynomials, etc.).
- Specification of models is rather simple and flexible. All functions for estimation of models, i.e., model* functions, are written with a common syntax in order to make the model specification task easier to the user. Besides, in the case of modelTF for TF or ARIMA model estimation and forecasting, the way the models are specified is in fact very similar to its analytical expression according to Eqs (1) and (2). For example, the MATLAB code for specifying an ARIMA(0, 1, 2) is ‘(1+ma1*B+ma2*B2)/(1-B)’, where ma1 and ma2 stand for two arbitrary names chosen to label the moving average coefficients and B is the back-shift operator.
  One advantage of this feature is that imposing constraints among parameters are straightforward. For example, if in the previous model for a given dataset the constraint ma1 = ma2 wanted to be imposed, the model code would be ‘(1+ma1*B+ma1*B2)/(1-B)’ instead, and only the ma1 parameter would be estimated.
- Either conditional or exact Maximum Likelihood (ML) estimation of ARIMA models are available, see e.g., [12]. Conditional ML is always used as a mean to obtain initial conditions for exact estimation, but it is convenient when the model involves very long time series or it is very complex, as is the case of models with multiple seasonal factors or many parameters.
- An algorithm for automatic identification of ARIMA models (function modelAUTO) is included, inspired in [5] and coded in the widespread forecast package in R. The procedure follows this reference except in the way differencing orders are identified. In particular, instead of relying on formal unit root tests, ECOTOOL selects difference orders by minimizing the variance of the resulting time series. This discrepancy is introduced due to many problems detected with formal unit root tests when applied to real time series. In addition, the automatic method is expanded to multi-seasonal models, making ECOTOOL the unique piece of software that implements this procedure, to the author knowledge.
- Automatic identification of four types of outliers are coded for ARIMA and TF models, following [21] and [4]. The types are additive, innovative, level shift and transitory change (coded in ECOTOOL as AO, IO, LS and TC). They are modeled as particular TFs applied to impulse dummy variables.
- ETS and UC models are estimated from their equivalent ‘reduced’ or ARIMA form, as in [22]. This allows to incorporate to these methods all the power ECOTOOL offers for modeling ARIMA processes. In particular, they may be estimated by exact ML, may include inputs as transfer functions and automatic detection of outliers may be carried out. All these are, once more, unique properties of ECOTOOL, as far as the author is concerned.
- The estimation output of any sort of models is rather exhaustive in tabular form. Such tables show parameter values with their standard errors and T tests, information criteria, correlation among parameters and, in the case of TF and ARIMA models, warnings about problems with unit roots in either numerator or denominator polynomials.

There are two functions that produce GUI interfaces that deserve special attention, namely toolTEST and toolFORECAST.

toolTEST is conceived as a friendly and exhaustive environment for descriptive statistics, as well as an identification tool and model validation tool of multivariate time series. When it is invoked, three menus unfold in a standard figure window in addition to the usual figure menus: i) a comprehensive ‘Tests’ menu, briefly explained below; ii) a ‘Series’ menu if the input is multivariate and allows to apply the tests to any individual and/or to all the time series at once; and iii) an ‘Options’ menu to deal with specific options for each item in the ‘Tests’ menu.

The ‘Tests’ menu offers a thorough combination of tabular and graphical information for many tests available. The following is a non-exhaustive list of such tests, classified in different categories:

- Descriptive information: time plots, box plots, scatter plots, descriptive statistics, histograms, formal Gaussianity tests [23, 24].
- Identification tools: univariate and multivariate sample autocorrelation and partial autocorrelation functions, Ljung-Box Q and Monti tests [25, 26], information criteria, Akaike’s, Schwarz, Hannan and Quin, [27–29], Granger causality tests based on VAR models [30].
- Constant mean and heteroscedasticity tests: CUSUM and CUSUMSQ tests [31], mean vs standard deviation scatter plots, formal ratio of variances heteroscedasticity test, Box-Cox transformation estimation [32, 33].
- Integration and cointegration tests: Dickey-Fuller and Perron unit root tests, Johansen cointegration tests [34–36].
- Non-linearity tests: [26, 37–39], Schwarz criterion on squares.
- Frequency domain tools: cumulative periodogram, smoothed or raw periodogram, AR-spectrum [16, 40].

The second useful GUI is the so called toolFORECAST, that is designed to show graphically the forecasting output of the model functions in ECOTOOL and to print out tables with plenty of error metrics (see examples in Case studies section).

Function toolFORECAST allows to plot one or several forecasts and forecasting errors for each time series in turn, selecting the output to be displayed interactively by menus. For long time series several pushbuttons permit to slide side-wise along the time series (as in toolTEST). Tabular results may be displayed in three different forms, i) actual values, forecasts, errors and error metrics for each time series and each forecasting step separately; ii) overall error metrics for one time series but many methods altogether to do fast comparisons; and iii) Diebold-Mariano test, sign test and Wilcoxon signed-rank test for testing statistical significant differences among several forecasting methods [41, 42].

## Case studies

The present section considers three case studies chosen to illustrate ECOTOOL working on real data. Not all the capabilities of the toolbox are shown in these examples, due to space restrictions. The documentation shows a wide range of thorough examples, run step-by-step with their respective coding, covering all the models and tools available in ECOTOOL. There, the implementation is shown deploying the code necessary to run the examples, together with the output produced.

The three forecasting cases shown below are designed following some common rules. They are rolling forecasting experiments in which the training in-sample data length, the testing out-of-sample data length, and the forecast origin are fixed initially depending on the properties of each dataset. The first round of forecasts with all the appropriate models is run and forecasted and the corresponding actual values stored. Then, the window is moved several samples ahead and all the forecasts are produced again with the models identified and estimated with the most recent information. This updating step is repeated to the end of the data.

This exhaustive evaluation of forecasting performance of each model is completed with the help of two error metrics that have proven very useful in many applications and are free from some inconveniences, namely the symmetric Mean Absolute Percentage Error (sMAPE) and the Mean Absolute Scaled Error (MASE), see Eqs (8) and (9) and [43, 44].

$$\begin{array}{c} {\text{sMAPE}}_{h}=h^{-1}\sum_{i=1}^{h}\frac{2|y_{i}-{\hat{y}}_{i}|}{|y_{i}|+|{\hat{y}}_{i}|}\times 100 \end{array}$$ (8)

$$\begin{array}{c} {\text{MASE}}_{h}=h^{-1}\sum_{i=1}^{h}\frac{|y_{i}-{\hat{y}}_{i}|}{{\left(n-s\right)}^{-1}\sum_{r=s+1}^{n}|y_{r}-y_{r-s}|} \end{array}$$ (9)

In Eqs (8) and (9) *y*_*t*_ and ${\hat{y}}_{t}$ are the actual and forecasted values at time *t*, respectively; *h* is the forecast horizon; *s* is the seasonal period of the data, if applicable, or just 1 if the data is not seasonal; and *n* is the number of observations in the fitting sample. The sMAPE metric avoids the distortions of the standard non-symmetric MAPE criterion and problems for values close to zero. The MASE metric compares the out-of-sample performance of the model with the in-sample performance of a simple seasonal RW (see Table 1), i.e. assuming that the model for the data is *y*_*t*_ = *y*_*t*−*s*_ + *a*_*t*_, with *a*_*t*_ white noise with zero mean, constant variance and serially independent.

### Forecasting analysis of Mauna Loa CO_2_ concentration data

**Fig 1:**
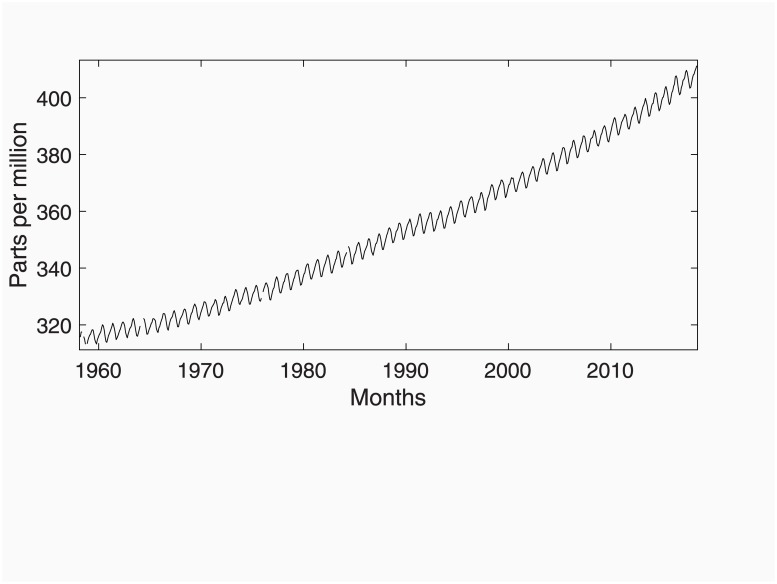
The Mauna Loa CO_2_ concentration data from March 1958 to May 2018.

The measured CO_2_ concentration at Mauna Loa is represented in Fig 1 (ftp://aftp.cmdl.noaa.gov/products/trends/co2/co2_mm_mlo.txt). It consists of sixty years of monthly data collected from March 1958 to May 2018. The data show a growing trend during the full period associated with long run emissions and a seasonal component rather stable related to the global net uptake and release of CO_2_ in summer and winter.

The upward trend and the seasonal variations may be also checked out by either the pseudo-periodogram (pseudo because the time series is not mean-stationary) or the AR pseudo-spectrum available in the toolTEST GUI tool (not shown here to save space). Clear peaks appear for zero frequency related to the trend and the seasonal periods 12 and 6 samples per cycle. The rest of harmonics do not show up.

To give the feeling of how the ECOTOOL code looks like, some coding is shown for this example. The next listing shows how to load the data, select the raw CO_2_ data (stored in the fifth column of the matrix data downloaded from the official web page), avoid the first two years of monthly observations, select 480 months after that and select two parameters that will be used later on, namely the number of forecasts (nofs) and the seasonal period (s). The last line is useful for those users who want to try the variance stabilizing Box-Cox transformation of the data (applied to the first 480 months). It can also be accessed by the ‘Box-Cox Transformation’ option in the ‘Tests’ menu of the toolTEST GUI. By this command the λ parameter of the transformation is estimated according to [33] and returns the transformed variable (ty) and λ (lambda).

load maunaLoa.data         % Load data

y = maunaLoa(25:504, 5);     % Select in-sample CO2

nofs = 24;             % Number of forecasts

s = 12;               % Seasonal period

[ty, lambda] = vboxcox(y);   % Box-Cox transform

The models used in this case study are all those available in ECOTOOL that may sensibly applied to this data. In particular:

- *Airline*: ARIMA(0, 1, 1) × (0, 1, 1)_12_ estimated with modelTF. The architecture of the model is maintained along the whole experiment, but the parameters are updated at each step.
- *Auto*: ARIMA automatically identified by modelAUTO. Both architecture and parameters are updated.
- *AR*: pure AR model automatically identified by a procedure similar to modelAUTO but truncated to avoid MA terms.
- *UC*: Unobserved components model composed of a Local Linear Trend with a trigonommetric seasonal component of period 12 samples per cycle. This is provided by modelUC with model option ‘LLT12’.
- *ETS*: additive Exponential Smoothing with level, slope and seasonal (modelES with model option ‘AA12’).
- *Naïve*: seasonal naïve, i.e. forecasts are taken as the last observation of the same season available (third column of output from modelNAIVE function).

The next listing shows how the previous models may be run for the first 480 months of the data (see details on how to use these functions in the toolbox documentation). Pure AR models are not included in the listing because they were produced with an ad-hoc particular function not included in ECOTOOL.

model = '(1+ma1*B)(1+ma12*B12)/(1-B)(1-B12)';

m1 = modelTF(ty, [], model, nofs);    % Airline model

m2 = modelAUTO(y, [], [1 s]);       % Auto ARIMA model

m3 = modelUC(ty, [], 'LLT12', nofs);   % UC model

m4 = modelETS(ty, [], 'AA12', nofs);   % ETS model

m5 = modelNAIVE(ty, nofs, s);       % Naive models

Mind that the syntax for all models are very similar and that the first input to modelAUTO is the time series without Box-Cox transformation because such change is tested inside this particular function. The output variables m1 to m5 are MATLAB structures with all the relevant information about the model output, like residuals, forecasts, etc.

**Fig 2:**
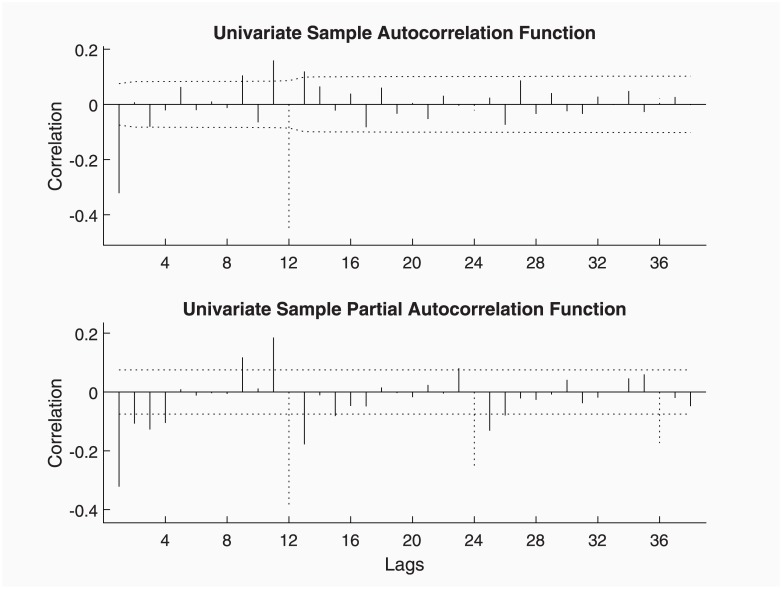
Simple and Partial Autocorrelation functions for the Mauna Loa differenced data. Dotted lines signal the seasonal lags.

The automatic ARIMA identification procedure implemented in ECOTOOL (by means of modelAUTO function) produces an ‘airline’ model, i.e. ARIMA(0, 1, 1) × (0, 1, 1)_12_. This model is perfectly supported by the Simple and Partial Autocorrelation functions for the differenced data shown in Fig 2. It certainly shows that the MA terms are essential to avoid over parameterisation if only AR terms are used, since finite order MA models are theoretically equivalent to infinite order AR models, as in [14].

Fig 2 is the result of the following code, though it can also be produced by toolTEST.

z = vdif(ty, [1 1], [1 s]);    % Difference series

vident(z);              % Identification

Calculating error metrics and statistical tests about forecast errors, and performing some graphical checks of forecasts are very simple with the help of the toolFORECAST function (actually a GUI). Next listing shows how to produce such outputs in the original scale of the variable. This requires to undo the Box-Cox transformation on the model forecasts, stored in field .py of all model structures (m1 to m5).

ACTUAL = maunaLoa(505:528, 5);

FORECASTS = vboxcoxinv([m1.py m2.py m3.py m4.py m5.py], lambda);

toolFORECAST(ACTUAL, FORECASTS)

The forecast exercise consists of a rolling out experiment in which the initial forecast origin is chosen at 480 observations from the beginning (March 1998) and the forecast horizon is 24 months ahead. Then, one month is added to the sample and the whole process is repeated to the end of the sample. Therefore, 220 total rounds of 24 months-ahead forecasts from all models are produced. The average forecasting performance of all models used are shown in Table 4. The table is divided in two parts reporting the average SMAPE and MASE metrics for each model for selected horizons ranging from 1 to 24 months.

**Table 4:** Average sMAPE and MASE metrics for different models on the Mauna Loa experiment.

	sMAPE						MASE					
Steps	Airline	Auto	AR	UC	ETS	Naïve	Airline	Auto	AR	UC	ETS	Naïve
1	0.065	0.067	0.072	0.068	0.067	0.549	0.166	0.170	0.181	0.171	0.170	1.386
2	0.072	0.074	0.077	0.077	0.075	0.549	0.182	0.187	0.196	0.194	0.189	1.386
3	0.078	0.079	0.083	0.084	0.081	0.549	0.197	0.199	0.210	0.213	0.205	1.387
4	0.082	0.083	0.088	0.090	0.086	0.550	0.209	0.212	0.224	0.227	0.218	1.388
5	0.086	0.087	0.092	0.095	0.091	0.550	0.218	0.222	0.234	0.240	0.229	1.388
6	0.089	0.091	0.095	0.099	0.094	0.550	0.226	0.231	0.242	0.250	0.238	1.388
1 year	0.107	0.107	0.112	0.114	0.118	0.547	0.273	0.274	0.287	0.290	0.299	1.381
2 years	0.139	0.137	0.144	0.145	0.168	0.818	0.355	0.351	0.370	0.371	0.426	2.068

Table 4 offers some interesting insights into the forecasting issue of the Mauna Loa data. Firstly, forecasts deteriorate with the horizon for all models, as expected. Secondly, all models show significant performance improvements over the Naïve, implying that they are really capturing the structure of the data beyond a naïve seasonal pattern. Thirdly, all errors are very small implying that the series can be forecast with great accuracy (take, for example, the Airline model that produces an average sMAPE of only 0.139% for 24 months ahead). Fourthly, no big differences in performance may be reported among models up to one year ahead with the exception of *Naïve*, though strictly speaking, *Airline* outperforms the rest at every single step. However, that is not the case for 24 steps ahead, where the best model is *Auto*, instead. Finally, *AR* model lies in a middle range, better than *UC* and *ETS*, while *ETS* deteriorates importantly for 24 months ahead.

**Fig 3:**
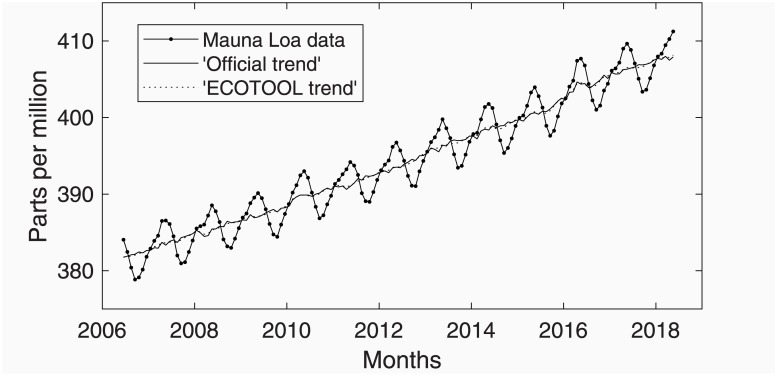
Detail of Mauna Loa data with the ‘official’ and modelUC trends.

The overall conclusion is that ECOTOOL provides a set of models rather reasonable for modelling and forecasting the Mauna Loa data. Moreover, the Unobserved Components may be used to extract the trend of the data (stored in field comp of structure m3) and compared to the ‘official’ one reported in the original web page. Fig 3 shows a detail of the data with the ‘official’ trend and the one estimated by modelUC. One single trend is visible because, though they are not exactly the same, both are consistent.

### Hourly electricity demand in Spain

**Fig 4:**
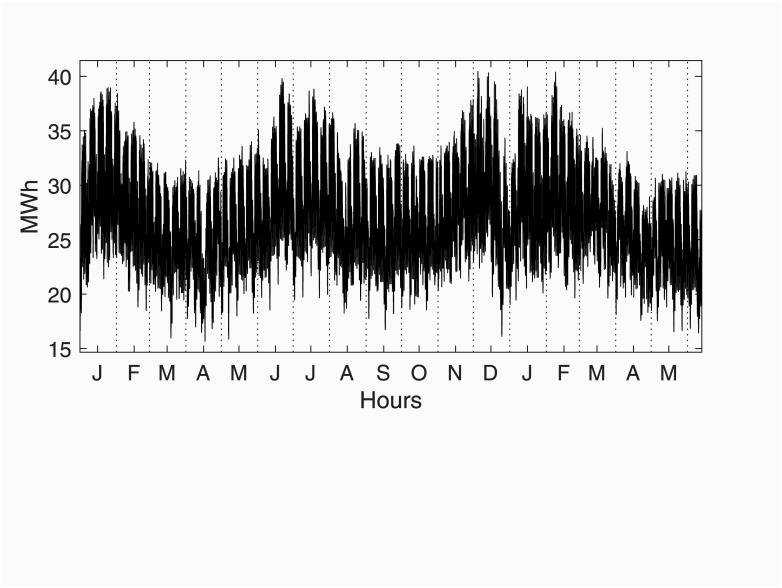
Spanish hourly electricity load demand from January 2017 to June 2018.

The data for the Spanish electricity demand used in this case study is publicly available at the Iberian Energy Market Operator web page (OMIE: http://www.omie.es). The data is continuously updated from 29 June 2001. Fig 4 shows a portion of such data, from 1 January 2017 to 12 June 2018.

These data are characterised by a number of periodic components superimposed that have to be dealt with, if a comprehensive model wants to be fitted. Firstly, the data exhibits a clear annual cycle with two peaks in winter and summer, respectively, closely related to temperatures. Secondly, a strong diurnal cycle, with different profiles depending on the season of the year. Thirdly, a weekly cycle is present with lower demand during weekends, mainly due to the absence of industrial activity. Finally, the data is affected by a number of special days, special events, moving festivals and holidays, etc.

In general, it is common to avoid modelling the year cycle for short term forecasting with hourly data, for several reasons: i) the most important drivers of the data in the short run are the daily and weekly cycle, while the annual cycle would become of paramount importance for longer horizons (from one week onwards); ii) it is parametrically unfeasible and much research should be conducted if trivial extensions of existing models want to be avoided. The problem is that the annual cycle holds 8,760 hours and a trivial extension of models to take into account the periodic behaviour would involve 4,380 harmonics. One way to tackle with these problems is with the aid of time aggregation techniques, e.g., [17], but they are rather specific methods that require specialised software, far beyond the scope of this paper, that focuses on presenting a toolbox for general use.

In this context is where ECOTOOL offers an important innovation, since automatic identification of ARIMA models is extended for these complex type of databases, namely the multi-seasonal ARIMA model, i.e., models that include as multiplicative seasonal factors as necessary. As far as the author is concerned, this is the first time that an automatic algorithm is developed for such complex cases. Certainly, in this case the model is composed of the multiplication of three ARMA factors, namely regular, daily and weekly seasonals. The general specification is in Eq (10), with the same nomenclature of Eq (1).

$$\begin{array}{c} z_{t}=\frac{\theta_{Q_{0}}\left(B\right)}{\phi_{P_{0}}\left(B\right)}\frac{\Theta_{Q_{1}}\left(B^{24}\right)}{\Phi_{P_{1}}\left(B^{24}\right)}\frac{\Theta_{Q_{2}}\left(B^{168}\right)}{\Phi_{P_{2}}\left(B^{168}\right)}a_{t} \end{array}$$ (10)

*θ*_*q*_(*B*)/*ϕ*_*p*_(*B*) is a ratio of polynomials in the back-shift operator of appropriate orders, respectively; $\Theta_{Q_{1}}\left(B^{24}\right)/\Phi_{P_{1}}\left(B^{24}\right)$ is similarly a ratio with orders *Q*_1_ and *P*_1_ in multiples of 24 hours per day; and $\Theta_{Q_{2}}\left(B^{168}\right)/\Phi_{P_{2}}\left(B^{168}\right)$ is similarly defined, though for 168 hours per week. The multi-seasonal extension of the ‘airline’ model used later is shown in Eq (11).

$$\begin{array}{c} y_{t}=\frac{\left(1+\theta B\right)}{\left(1-B\right)}\frac{\left(1+\Theta_{24}B^{24}\right)}{\left(1-B^{24}\right)}\frac{\left(1+\Theta_{168}B^{168}\right)}{\left(1-B^{168}\right)}a_{t} \end{array}$$ (11)

*y*_*t*_ in Eq (11) is the undifferenced time series because the differences are included explicitly in the model denominators.

**Fig 5:**
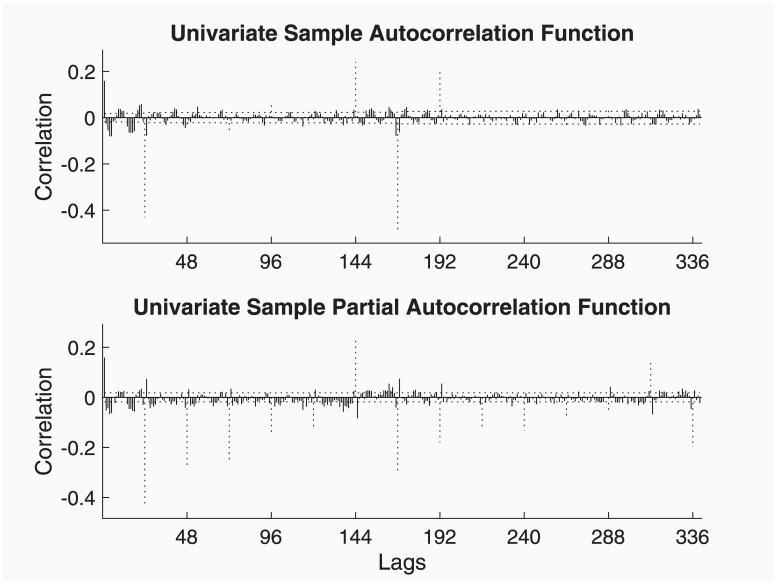
Simple and Partial Autocorrelation functions for the Spanish load demand data. Dotted lines signal daily lags.

The automatic identification applied to these data suggests that daily differencing is not necessary, implying that the ‘airline’ model in Eq (11) is strictly wrongly specified because of over-differentiation. However, manual identification of demand series imposing the differences implied by the ‘airline’ model (i.e., $\left(1-B\right)\left(1-B^{24}\right)\left(1-B^{168}\right){\text{demand}}_{t}$) produces a clear evidence in favour of the airline model except for the non-seasonal part of the correlogram (lags 1 to 11, see Fig 5). Therefore, the airline model will be kept as a benchmark to compare with.

The models trained are: i) a weekly Naïve of period 168 hours per cycle as a bottom benchmark (*Naïve*, daily naïve with period 24 was also tried, but was discarded because the results were systematically worse); ii) multi-seasonal airline in Eq (11) as a more sophisticated benchmark (*Airline*); iii) multi-seasonal model in Eq (10) automatically identified with modelAUTO (*Auto*); and iv) multi-seasonal AR model identified in a similar manner (*AR*).

All models were estimated and used to forecast a week ahead along a full year (from July 2017 to June 2018) with a rolling forecast origin every 6 hours and samples of 8 weeks length. Thence, 1,460 rounds of 168 hours-ahead forecasts were calculated with each model. The window size (8 weeks) allow the models to adapt for the changing profile of the seasonal components over the year. A full year of data was reserved as the test set to give a better overall idea of forecasting performance, since such performance varies with the season of the year.

Results for sMAPE and MASE metrics are shown in Table 5 for a selection of forecast horizons. Some relevant observations follow. Firstly, the forecast performance deteriorates with the forecast horizon for all methods. Secondly, the *Auto* method is the best for all horizons when compared with *Airline*, meaning that the automatic identification implemented in ECOTOOL makes sense in terms of forecasting performance. Thirdly, *Auto* is better than *AR* as well, implying that including moving average terms in the models pays back in terms of forecasting performance. Finally, a striking result is that the deterioration of the *Naïve* model is much slower than in the rest of cases (though its performance for shorter horizons is rather poor), but it is the best options for the long horizons, for 6 and above days.

**Table 5:** Average sMAPE and MASE metrics for different models on electricity demand data.

	sMAPE				MASE			
Steps	Airline	Auto	AR	Naïve	Airline	Auto	AR	Naïve
1	1.945	1.910	2.101	6.061	0.236	0.232	0.255	0.774
2	2.347	2.316	2.563	6.130	0.285	0.283	0.312	0.786
3	2.607	2.575	2.844	6.200	0.320	0.317	0.350	0.798
4	2.797	2.753	3.037	6.251	0.346	0.341	0.377	0.808
5	2.928	2.863	3.163	6.276	0.364	0.356	0.395	0.812
6	3.021	2.934	3.252	6.289	0.377	0.366	0.407	0.812
7	3.135	3.032	3.382	6.257	0.391	0.378	0.423	0.806
8	3.268	3.151	3.529	6.250	0.408	0.393	0.442	0.805
9	3.398	3.260	3.666	6.260	0.424	0.407	0.460	0.807
12	3.672	3.488	3.952	6.291	0.460	0.437	0.498	0.812
1 day	4.399	4.084	4.734	6.289	0.557	0.516	0.602	0.812
2 days	5.400	4.860	5.607	6.282	0.688	0.616	0.718	0.811
3 days	6.216	5.369	6.205	6.282	0.795	0.682	0.797	0.811
4 days	6.920	5.737	6.663	6.294	0.888	0.730	0.860	0.813
5 days	7.634	6.096	7.074	6.303	0.982	0.778	0.917	0.814
6 days	8.383	6.436	7.483	6.309	1.080	0.823	0.974	0.815
7 days	9.106	6.711	7.819	6.314	1.175	0.859	1.024	0.816

This latter observation means that standard time series models focus on short-term horizons and more sophisticated extensions should be provided for longer horizons. One clear extension would be to add the annual cycle into the models, since it could be the case that for horizons long enough the lack of a annual cycle starts to be felt.

One final point worth considering is that the optimal forecasts of the Mauna Loa data in the previous case study are systematically much more accurate than the electricity demand forecasts, because of a much greater level of uncertainty in the latter case. This point may be checked by comparing Table 4 with the appropriate rows in Table 5, bearing in mind that 12 and 24 hours ahead in Table 5 corresponds to 1 and 2 years in Table 4, respectively. The sMAPE for electricity demand 24 steps ahead is about 30 times the sMAPE for the CO_2_ concentration data.

### Global Horizontal Irradiation forecasting at a photovoltaic plant in Ciudad Real, Spain

**Fig 6:**
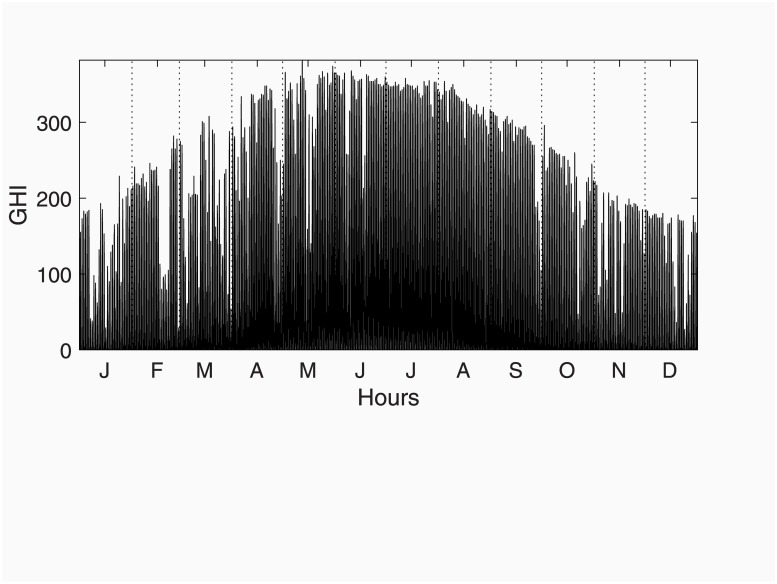
A full year of GHI data at a photovoltaic plant in Ciudad Real, Spain.

This case study evaluates the forecasting performance of the models implemented in ECOTOOL when applied to the Global Horizontal Irradiation (GHI) data provided by the Spanish Meteorological Estate Agency (AEMET) weather station located at Ciudad Real, Spain. The original dataset consisted of 18 years of GHI hourly observations. Fig 6 shows an overview of the last year of data that shows clearly the annual cycle and the variability among different days, sometimes weeks, depending mainly on the cloud cover.

One typical feature of the data is that GHI drops down to zero every night at different times within the day depending on the sunrise and sunset times. Consequently, the time series contains numerous zeros deterministically located along the year. In winter there are just 10 sun hours, while in summer the Sun shines for up to 16 hours. An efficient way to deal with this singularity of the data is removing such zeros before the modelling stage and inserting them back to build the final forecast for full days. In this way, at each forecast origin the periodicity of the data is different, depending on the time of the year.

**Fig 7:**
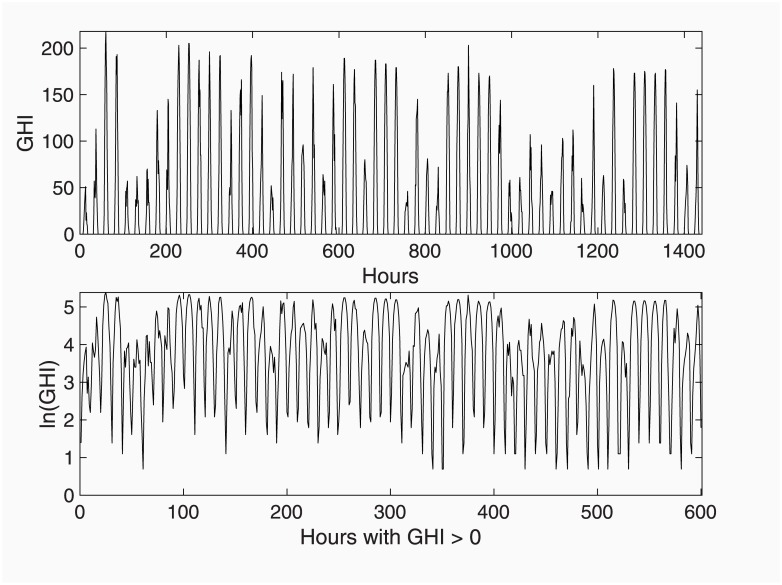
Two months of original GHI data (top panel) and the data after transformation with Box-Cox and removal of night zero values.

Due to this peculiarity, the data is rather heteroscedastic along the year. This problem may be alleviated by the Box-Cox transformation, that in ECOTOOL is implemented in the function vboxcox (that may be run directly or by a menu option within toolTEST), in which the optimal lambda is estimated following the model-independent approach by [33]. Lambda turned up to be close to 0 in most cases, meaning that the optimal transformation is the natural logarithm. Fig 7 illustrates the convenience of these two transformations. Top panel shows two months of the original data, while the bottom panel shows the data in logs and after zero-removal. The sample length is drastically reduced (only 10 samples per day out of 24 remained in the case shown) and the natural logarithm transformation renders a time series with proper statistical properties, at least regarding homoskedasticity.

This case, unlike electricity demand, is not multi-seasonal, since only a diurnal period is observed on top of an annual cycle. The annual cycle is ignored because models used are sensible strictly for short run forecasting (see discussion on this issue in the previous case study). Then, the methods used in this case are the ones already used in the Mauna Loa case, but with time varying periods due to the zero values removal. More specifically the models are ARIMA *Airline*; ARIMA *Auto* identified by means of modelAUTO; automatic *AR* models; *UC* model; *ETS* and seasonal *Naïve* of period 24 hours.

To illustrate ECOTOOL working on this data, the rolling experiment is conducted by selecting samples of 2 months of data previous to each forecasting origin. One week ahead of data is forecast at each step and the forecast origin is moved 6 hours forward. The evaluation is repeated along a full year of data, i.e., 1,460 total runs of each model.

sMAPE metrics cannot be computed in this case due to the presence of simultaneous zeros in data and forecasts. Indeed, inspection of Eq (8) shows this is one of few particular cases where sMAPE is not defined because both forecasts and actual values are zero. Cases like this highlights the utility of other metrics, like MASE that may be still be computed.

Results are reported in Table 6, showing clearly that the GHI data is less forecastable than the previous cases. The main reason for this is that all the MASE measurements are much bigger now. As an example, the *Auto* model renders MASE values that are about twice the electricity case and almost four times the Mauna Loa case for 12 steps ahead forecasts. But the main reason is that the *Naïve* model, i.e., projecting as forecasts into the future just the last seasonal cycle available, is rather good. Indeed, for horizons longer than 9 hours ahead, the *Naïve* model is the absolute winner, and it is the best than most of them for horizons longer than 6 hours ahead.

**Table 6:** MASE metrics for different models on a GHI data from a photovoltaic plan in Spain.

Steps	Airline	Auto	AR	UC	ETS	Naive
1	0.578	0.582	0.648	0.586	0.753	0.924
2	0.718	0.712	0.793	0.744	0.917	1.001
3	0.847	0.821	0.924	0.926	1.108	1.049
4	0.957	0.905	1.026	1.127	1.304	1.063
5	1.036	0.953	1.101	1.290	1.462	1.054
6	1.090	0.972	1.143	1.407	1.577	1.034
7	1.135	0.991	1.177	1.485	1.659	1.019
8	1.184	1.015	1.213	1.544	1.739	1.027
9	1.235	1.042	1.261	1.590	1.812	1.040
12	1.306	1.076	1.329	1.670	1.916	1.035
1 day	1.346	1.110	1.403	1.653	1.855	1.036
2 days	1.484	1.191	1.623	1.700	1.894	1.098
3 days	1.602	1.257	1.910	1.724	1.916	1.144
4 days	1.731	1.328	2.314	1.754	1.943	1.192
5 days	1.860	1.397	2.911	1.777	1.961	1.222
6 days	1.985	1.463	3.867	1.793	1.973	1.247
7 days	2.112	1.526	5.608	1.803	1.982	1.268

But still, this confusing evidence should not distract from other type of evidence. Firstly, for horizons shorter than 9 hours the best method is *Auto*. Secondly, the performance of *ETS* is rather poor, since is better than *Naïve* only for 1 and 2 hours ahead. Thirdly, *AR* is more accurate than *UC* and *ETS* for short horizons, but it deteriorates rather badly for longer horizons. Finally, *Airline* and *Auto* outperforms *AR* for any forecast horizon.

Putting together all this evidence, several conclusions follow:

- Forecasting GHI data is rather difficult because of its inherent volatility. Improving simple models for horizons longer than 9 hours ahead may require a lot of modelling effort. The improvements may be more related to the use of inputs (like cloud cover) than to other methods closer to Machine Learning that usually concentrate on very short forecast horizons. However, predicting cloud cover to use them as an input to GHI may be more complex than predicting GHI itself.
- Pure AR models are sub-optimal, especially bad in this case study. If ARIMA models want to be used, there is no doubt that MA terms are very helpful in relation to forecasting accuracy, in contrast to the view of [14].
- The automatic identification of ARIMA models implemented in ECOTOOL produces models that outperform the rest.

## Conclusions

This paper has introduced ECOTOOL, a toolbox intended mainly for professional practitioners, academic researchers, students, and anyone involved in the analysis of time series, forecasting or signal processing. ECOTOOL is composed of a number of powerful functions to estimate a wide range of models in a rather user friendly manner; with abundant tools for identification, validation and graphical representation of results.

The main methods implemented are ARIMA, Exponential Smoothing, Unobserved Components, ARX, ARMAX, Transfer Function, Distributed Lag models and VARX. Several properties are the salient features of the toolbox, e.g. it is user-oriented; just a few MATLAB functions are enough to carry out a complete analysis of time series; model specification is rather simple and flexible; several estimation methods are implemented; automatic detection and estimation of four types of outliers is implemented; the toolbox is very robust making it useful in long automatic experiments in programming environments.

The toolbox also provides a wide range of descriptive information of the data, both graphically and in tabular format; standard and not so standard identification tools; formal and visual tests for gaussianity, independence, causality, heteroscedasticity, non-linearity, unit root and cointegration; spectral tools; tests on forecasting performance; etc.

Though many of the procedures implemented may be found in other software packages, some of them are exclusive to ECOTOOL, to the author knowledge. It is the case of the automatic identification of multi seasonal ARIMA models, automatic detection of outliers in Unobserved Components and Exponential Smoothing models, and the possibility of estimating Unobserved Components and Exponential Smoothing models by Exact Maximum Likelihood adding inputs specified as dynamic transfer functions.

The toolbox is shown working on three case studies, in which several methods are tested on forecasting time series with different sampling interval and degrees of complexity, namely the monthly CO_2_ concentration data at Mauna Loa, hourly electricity demand in Spain, and hourly global horizontal irradiation at a photovoltaic plant in Ciudad Real, Spain.

Results show clearly that forecastability depends strongly on the case, being the global irradiation the worst case to forecast. In all cases, the automatic procedure of identification of ARIMA models shows great potentiallity as a general tool in forecasting tasks and including moving average terms in ARIMA models increases forecast accuracy.
